# COVID-19: A Review on Diagnosis, Treatment, and Prophylaxis

**DOI:** 10.3390/ijms21145145

**Published:** 2020-07-21

**Authors:** Alessandra Fierabracci, Andrea Arena, Paolo Rossi

**Affiliations:** Infectivology and Clinical Trials Research Department, Children’s Hospital Bambino Gesù, 00146 Rome, Italy; andrea.arena@opbg.net (A.A.); paolo.rossi@opbg.net (P.R.)

**Keywords:** SARS-CoV2 infection, prevention, treatment, immune system, outbreak

## Abstract

Coronavirus 2 (CoV) Severe Acute Respiratory Syndrome (SARS-CoV2) is causing a highly infectious pandemic pneumonia. Coronaviruses are positive sense single-stranded RNA viruses that infect several animal species, causing symptoms that range from those similar to the common cold to severe respiratory syndrome. The Angiotensin Converting Enzyme 2 (ACE2) is the SARS-CoV2 functional receptor. Measures are currently undertaken worldwide to control the infection to avoid disruption of the social and economic equilibrium, especially in countries with poor healthcare resources. In a guarded optimistic view, we hope that the undertaken preventive and treatment measures will at least contribute to contain viral diffusion, attenuate activity, or even eliminate SARS-CoV2. In this review, we discuss emerging perspectives for prevention/treatment of COVID-19 infection. In addition to vaccines under development, passive immunization is an open opportunity since patients develop neutralizing antibodies. A full spectrum of potential drugs for COVID-19 infections could in turn affect virus binding or enzymatic activities involved in viral replication and transcription. Furthermore, clinical trials are currently evaluating the safety and efficacy of anti-inflammatory drugs, such as tocilizumab. Bioinformatics may allow characterization of specific CD8+ and CD4+ T cell responses; thus, CoV2 T cells’ frequency can be correlated with the disease severity and outcome. Combinatorial antibody phage display may be empowered to identify the immune repertoire of CoV2-specific neutralizing antibodies.

## 1. Introduction

The 2019 novel coronavirus disease (COVID-19) is a highly infectious pneumonia recently recognized as “pandemic” by the WHO [1]. Mysterious COVID-19 pneumonia was first reported in Wuhan, Hubei Province, China, in December 2019, followed by outbreaks all over the world [1,2].

**Figure 1:**
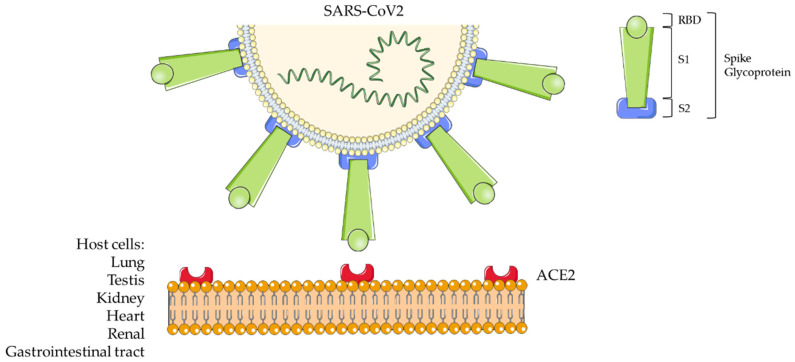
Severe Acute Respiratory Syndrome-Coronavirus 2 (SARS-CoV2) structure and host cell interaction.

COVID-19 has been also named SARS (Severe Acute Respiratory Syndrome)-Coronavirus (CoV)2 by the Coronavirus Study Group (CSG) of the International Committee on Taxonomy of Viruses (ICTV). The novel virus is indeed part of the taxonomic Coronaviridae family (subfamily Coronavirinae) [3]. Coronaviruses (CoVs) are positive sense single-stranded RNA viruses. They infect a wide variety of animal species, causing a wide range of symptoms, such as those similar to the common cold to severe/fatal respiratory syndrome [1]. Chinese scientists isolated the virus from biological samples of affected patients during the recent China outbreak and defined its genetic code [4]: GenBank Database MN908947.3. Coronaviruses comprise a large family of viruses being classified within the α, β, γ, and δ genera [3,5]. CoV2 is a representative betacoronavirus sharing nucleic acid sequence similarity with SARS-CoV and the Middle East Respiratory Syndrome (MERS)-CoV. The crown-like morphology observed in electron microscopy specimens is due to the presence of spike (S) glycoproteins on the viral surface [6]. These proteins are constituted by an ectodomain with a receptor-binding unit called S1 and a membrane-fusion unit called S2 (Figure 1). The S1 unit must bind a cell surface receptor through a receptor-binding domain (RBD) to invade host cells. Instead, S2 contributes to fuse the host cellular and viral membranes; thus, the viral nuclei acid can penetrate into the host cells. The Angiotensin Converting Enzyme 2 (ACE2) has been identified as a functional receptor for SARS-CoV [7,8] (Figure 1).

Furthermore, the viral spike glycoproteins include a transmembrane anchor and a short intracellular tail at C-terminal [5]. ACE2 is an exopeptidase that facilitates the conversion of Angiotensin I to the nonapeptide Angiotensin 1-9 or of Angiotensin II to Angiotensin 1-7 [6]. Residue phenylalanine F486 in the flexible loop of RBD has relevance in the interaction with ACE2 since it penetrates into its hydrophobic pocket [6].

ACE2 is widely expressed from fish to amphibians, birds, and reptiles to mammals; all these animal species can be natural hosts of SARS-CoV2. Since ACE2 is highly expressed in the lung, intestine, testis, and kidney, in addition to salivary droplets, other routes of transmission of the viral infection are possible [6]. Indeed, recent studies provide morphological and/or proteomic evidence of SARS-CoV2 infection and host-viral protein interaction from human colon epithelial (Caco-2), lung (A549-ACE2), monkey (Vero E6) kidney cell lines [9], and endothelial glomerular capillary loops from patients [10]. The highest levels of SARS-CoV2 RNA copies/cell were detected in human autoptic samples from the respiratory tract; lower levels were assayed in kidney, liver, heart, brain, and blood cells, suggesting a broad organotropism [11]. Further cytopathic effects of SARS-CoV2 were detected in human induced-pluripotent stem cells (iPSC)-derived cardiomyocytes [12].

Wang et al. (2020) [13] first described the epidemiological, clinical, and laboratory characteristics of COVID-19 infectious syndrome. From a pathogenetic point of view, the infection begins when the virus passes the nasal and larynx mucosa to enter the respiratory tract and reach the lung. Early symptoms include fever and cough [14]. From the lung, the virus enters the peripheral blood, causing viremia and targeting organs that express ACE2, including the heart and renal and gastrointestinal tract [14]. This explains why the virus is found in the fecal samples [13,15]. Usually 8 days occur from the symptoms’ onset to the respiratory syndrome, leading to exacerbation until 14 days from the onset. Blood cell counts in peripheral blood is normal or slightly low at the beginning, with eventual lymphopenia [13]; this may affect antibody production. If in the acute pneumonia phase, the immune system is effective, and the patient is not affected by underlying conditions, the virus is suppressed, and recovery occurs. Instead, if the patient is in advanced age, the disease will become severe. Non-survivors were found to exhibit higher neutrophils counts, D-dimer levels, blood urea nitrogen, creatinine, and inflammatory cytokines than survivors [14].

Considering the impact of COVID-19 ongoing global public health emergency, measures to reduce person-to-person transmission of the virus, thus containing its spread, are currently undertaken all over the world [16,17].

At present, vaccines are under development, and there are no specific antiviral treatments to defeat COVID-19 infection in humans and other animal species [18,19,20,21]. Thus, it is of upmost importance to identify and unravel treatments that may cure the disease in infected subjects and eradicate it in affected populations.

In this review, we discuss emerging perspectives for the appropriate preventive and treatment strategies to tackle COVID-19 infection. These include vaccine development based on definition of immunodominant viral epitopes, passive immunization, several anti-viral therapeutics, and anti-inflammatories. We also highlight tools that may elucidate mechanisms governing viral infection and clearance at the different stages of disease and advance effective immunotherapies based on monoclonal antibodies discovery.

## 2. Diagnostics

In the past, MERS and SARS-CoV have caused large-scale epidemics; however, from recent epidemiological data, SARS-CoV2 presents a higher rate of transmission; on average, 2 or 3 people become infected from one already infected person [22]. Molecular assays based on reverse real-time PCR (rRT-PCR) are today the gold standard for screening and diagnosis of COVID-19 infection in early stage, verifying its course, and estimating the extent of viremia. The virus can be detected on a variety of clinical specimens from bronchoalveolar lavage fluid, saliva, pharyngeal and nasal swabs, stool, or peripheral blood [22,23]. Recently, as a novel nucleic acid amplification technique, Loop-mediated isothermal amplification (LAMP) reaction was developed. This method, under isothermal conditions, rapidly amplifies DNA with high specificity and efficiency. It is based on the use of four specially designed primers (complementary to a total of six distinct sequences on target DNA) and of the enzyme DNA polymerase, which, having strand displacement activity, amplifies target DNA up to 10^9^ copies in less than an hour at 65 °C [24].

Serological immunoassays are currently used to estimate seroconversion in seroprevalence surveys in people suspected of current or previous SARS CoV2 infection through anti-viral IgG and IgM immunoglobulins testing. Accuracy of antibodies detection is assessed by enzyme-linked immunosorbent, chemiluminescence, and flow laminar immunoassays. IgGs persist for the longest time and may reflect a longer-time immunity, although it is the last to rise after infection since positivity occurs between 13–21 days. Reported time to IgM positivity ranges from 5 to 10 days following disease onset but declines soon after an infection is cleared [25]. Combining serology with PCR results can enhance sensitivity and accuracy of early diagnosis of CoV2 infection.

Other abnormalities were detected in COVID-19 patients, such as lymphopenia, increased levels of C reactive protein (CRP), lactate dehydrogenase (LDH), erythrocyte sedimentation rate (ESR), and D-dimer [26]. In addition to low levels of serum albumin, increased values of total bilirubin, creatinine, cardiac troponins, aspartate aminotransferase (AST), alanine aminotransferase (ALT), prothrombin time (PT), and procalcitonin were detected and found of prognostic significance [26]. Hematological parameters, such as leukocytosis, neutrophilia (indicative of bacterial superinfection), and lymphopenia (indicative of reduced immune response to the virus), are predictors of progression toward severe respiratory distress in COVID-19 infected people. Lippi et al. (2020) [27] discovered significantly increased values of MDW (monocyte volume distribution width—DxH 900 hematology analyzer, Beckman Coulter, Brea, CA, USA) in all COVID-19 patients, especially with severe manifestations. As for severe and/or systemic infectious diseases, consumption (disseminated) coagulopathy may be one of the most severe complications of patients with COVID-19 as assessed by hemostasis testing PT and D-dimer values [28,29].

## 3. Treatment

### 3.1. Passive Immunization

It is known that SARS patients develop strong neutralizing antibody responses against the virus [30]. Indeed, a meta-analysis demonstrated significant reduction in mortality and viral load, thus effectiveness of convalescent plasma (CP) immunotherapy for the treatment of SARS-CoV and MERS-CoV [31]. In principle, this may represent a tool to increase protection against CoVs by binding S-proteins immunogenic parts thus neutralizing their function. In the course of the present COVID-19 pandemic, recent attempts suggest that, indeed, early administration of CP from recovered patients at high titers of neutralizing antibodies can likely reduce the viral load and disease mortality. CP transfusion may affect COVID-19 pathogenesis through direct neutralization of the virus, controlling a cytokine storm, Th1/Th17, and complement cascade activation and immunomodulating the hypercoagulable state [32,33]. In the study by Duan et al. (2020) [34], one dose of 200 mL of CP with neutralizing antibody titers above 1:640 was administered to 10 patients. In addition, patients received maximal supportive care and anti-viral agents. In this initial trial, the primary endpoint was to verify safety of CP infusion; the second endpoint was improvement of clinical symptoms and laboratory parameters within 3 days after treatment. No severe side effects were reported. No specific virus was detected in CP before transfusion; indeed, plasma was subjected to methylene blue photochemistry to inactivate residual viral particles. Patients did not develop transfusion-related lung damage. Further, there was no antibody-dependent infection enhancement that may occur when subneutralizing concentrations of antibodies are transfused through suppression of innate mechanism of defense with the consequence of intracellular growth of the virus [35]. Small group studies were also reported from Chinese clinical Centers [36,37,38]. An investigation on 25 patients recruited at the University of Texas (Houston, TX, USA) confirmed CP safety; however, it underlined the difficulty of analyzing data due to confounding variables of other treatment regimens, such as antivirals and anti-inflammatories administration [39]. Nevertheless, from the initial studies of CP transfusion, optimal dosing, timing and clinical benefits of this approach will require further evaluation in larger well-controlled trials. Investigations have been undertaken to explore the use of neutralizing human monoclonal antibodies against CoV2 generated from B cells of convalescent patients (NCT04354766). One ongoing clinical trial is recruiting patients in Shanghai (China), but, to our knowledge, no relevant results were announced yet (NCT04292340) for anti-SARS-CoV2 CP [37]. In another trial, carried out in China by recruiting patients with severe or life-threatening COVID-19 syndrome from 7 medical centers, CP therapy added to standard treatment did not significantly improve timing to clinical amelioration within 28 days compared to standard treatment. Interpretation of results may have been affected by early termination of the trials originally recruiting 103 participants [40]. A trial was recently started at the Columbia University Irving Medical Center in New York (NCT04359810, New York, NY, USA) to assess CP efficacy on a cohort of 129 enrolled patients [41]. An interventional proof of concept single-arm trial to produce hyperimmune plasma from convalescent donors to be administered to critical COVID-19 patients was started in Italy in March 2020 (NCT04321421), with the limited recruitment of 43 patients [42]. On a general ground, the use of human products, such as human immunoglobulin administration, is, however, associated with increased risk of same day thrombotic events [32,43]. Certainly, the use of CP with high neutralizing antibody titers should follow precise ethical and controlled measures of selection, especially in absence of knowledge of the basic biology of COVID-19, including its variability and mutation capacity [43].

A potential alternative challenge is to directly administrate neutralizing monoclonal antibodies (mAbs) to subjects encountering the infection. Generated mAbs can interrupt any stage of the viral lifecycle or the host cell receptor proteins, thereby affecting virus binding, attachment on the cell surface, and entry into the host cell [44]. Similarities between RBD of different SARS-CoVs might justify the neutralization activity of SARS-like CoV neutralizing mAbs against SARS-CoV2. This hypothesis needs to be confirmed by a comparative analysis of the RBD sequence of SARS-CoV2 and SARS-CoV to verify that RBD specific mAbs can potentially be of efficacy and thus be recommended for evaluation in clinical trials. Combination therapy of remdesivir (GS-5734) [45] with mAbs could be an option to be experimented [46]. Remdesivir is an experimental drug for the treatment of Ebola virus infection that was effective for the treatment of MERS and SARS-CoV in animal models.

Another possible route of investigation is the safe passive immunization of exposed individuals and patients throughout the use of human single chain antibodies (Hu-scFvs) or humanized nanobodies (single-domain antibodies (sdAb)). These molecules or transbodies directed to CoV intracellular pathways/non-structural proteins can go across the membrane of virally infected cells thus leading to block viral replication and transcription. Transbodies have already being experimented for the treatment of other infections, including those caused by influenza, hepatitis C, Ebola, and Dengue viruses [47].

The future use of mAbs could overcome many drawbacks that are associated with the administration of plasma and intravenous immunoglobulin preparations, as above outlined, especially in terms of specificity, safety, purity, and low risk of transmission from blood-borne pathogens [44]. We need, however, to underline that in spite of the promising results obtained with mAbs in neutralizing SARS-CoV and MERS-CoV infections their generation and subsequent large-scale production could be time consuming, laborious, and expensive. This limits their applicability against emerging pathogens, such as SARS-CoV2, in respect to CP transfusion [44].

### 3.2. Therapeutics

Management of COVID-19 infection aims first to ameliorate clinical manifestations and to provide supportive care [48]. Nevertheless, long-term experimentation may be necessary for researchers to develop, produce, standardize, assess, and commercialize novel drugs specific to the novel virus.

To date, there is a full spectrum of potential drugs for COVID-19 infections that affects virus binding or enzymatic activities involved in viral replication and transcription. Essential viral proteins may be target of small molecule inhibitors i.e., helicase, proteases, inhibitors of host cell proteases, and endocytosis and fusion core blockers. Furthermore, anti-sense RNA (siRNAs), ribozyme, neutralizing antibodies, mAbs binding host receptor or that affect S1 RBD interaction, antiviral peptides that act on S2, and other natural products can be exploited [49,50].

Experimentation of drugs for COVID-19 management should certainly start to screen for efficacy and safety of clinically assessed MERS- and/or SARS-CoVs inhibitors, although some precautions derive from the still unexplored properties of the novel COVID-19 virus [16]. Indeed, the RNA-dependent RNA polymerase (RdRp) sequence of SARS-CoV2 shares 96% identity to SARS-CoV [51]. This, in particular, suggests that drugs targeting SARS-CoV RdRp might exhibit similar efficacy for SARS-CoV2 RdRp [51].

Clinical trials are required in particular for confirming safety and efficacy for COVID-19 RNA synthesis inhibitors (like 3TC, TDF), remdesivir, neuraminidase inhibitors, peptide (EK1), anti-inflammatory drugs, and arbidol [52]. In vitro and in vivo studies have shown anti-CoV activities of molecules that affect the S1 protein as either S-protein inhibitors, S cleavage inhibitors and RBD–ACE2 blockers. In this regard, phase III trials are ongoing with remdesivir, lopinavir, oseltamivir, darunavir, the HIV (Human Immunodeficiency Virus) protease inhibitor ASC09F, ritonavir, and cobicistat [52]. There is, so far, a lack of supportive evidence of safety and efficacy of Chinese traditional medicine products already employed to treat other CoV infections [16,53].

Of note, binding of the S1 COVID-19 protein to ACE2 has an effect on the renin–angiotensin system (RAS), leading to possible exacerbation of severe pneumonia. Currently, ACE1 and angiotensin type-1 receptor (AT1R) inhibitors are believed to reduce pulmonary inflammation, thus reducing the mortality rate [16,54].

Remarkably, antiviral drugs already experimented for CoV infections can potentially treat COVID-19. The use of HIV protease inhibitor lopinavir in association with ritonavir in recent Chinese trials on SARS-CoV2 patients requires further evaluation and investigation [55,56]. These antiviral molecules are of known pharmacokinetic and pharmacodynamic properties, dosing regimens, stability, and adverse effects and are easily available [16,20].

Of note, cytokines play important roles in the immunopathology of viral infections since innate immunity is the first line defense against viruses. Dysregulated proinflammatory immune responses cause the ‘cytokine storm’ in the pathogenesis of CoV2 infection. This is closely related to the development and progression of the Acute-Respiratory Disease Syndrome (ARDS) or extrapulmonary multiorgan failure, leading to infection exacerbation or death. Therefore, immunomodulators and cytokine antagonists represent a potential effective strategy. In this regard, evidences were produced on the efficacy of anti-inflammatory drug tocilizumab to ensure the possibility to timely control the cytokine storm at the early stage to improve success rate of treatment and reduce mortality rate. Tocilizumab is a recombinant humanized monoclonal antibody raised to both soluble IL-6 receptor (sIL-6R) and bound to the membrane (mIL-6R) [57]. This drug is indicated for the treatment of severe chronic inflammatory disorders, such as rheumatoid arthritis, systemic juvenile idiopathic arthritis, and juvenile idiopathic polyarthritis [57]. It also finds application for the management of severe or life-threatening cytokine release syndrome (CRS) that can be elicited by chimeric antigen receptor T-cell (CAR) T therapeutic approaches. Chinese researchers reported preliminary data on efficacy of tocilizumab (at the expected dose of 400 mg iv for CRS treatment) in 21 patients with severe or critical COVID-19 pneumonia [58]. They observed reduction of oxygen requirement, resolution of CT (spiral computerized tomography) lesions, normalization of lymphocyte count, reduction of C-reactive protein levels, and hospital discharge with an average hospitalization duration of 13.5 days. In Italy, AIFA (Agenzia Italiana del Farmaco) recently started a protocol for evaluating the efficacy of tocilizumab in treating respiratory distress in COVID-19 patients (EudraCT Number 2020-001110-38) (Table 1).

Regarding the antiviral activity of Type 1 interferons (IFN-1), their beneficial effects are at an early stage of infection. At a later stage, their administration may worsen the cytokine storm and exacerbate inflammation [59]. Type 1 interferons designate a group of cytokines, including α, β, ε, ω, and κ subtypes secreted by various cell types, especially plasmacytoid dendritic cells upon recognition by pattern recognition receptors (PRR). Since Type 1 interferons play a role in antiviral response and low or delayed IFN response associated with poor outcome, IFN α-β have emerged as potentially effective drugs against CoV2. Type 1 interferons are most often used in combination with other antiviral drugs, such as lopinavir, ritonavir, or ribavirin [60]. In the study by Hung et al. (2020) [60], the efficacy and safety of IFN β1b, lopinavir, ritonavir, and ribavirin was assessed in a multicenter, prospective, open-label, randomized, phase II trial in adults. Zhou et al. (2020) [61] compared 77 adults affected by COVID-19 treated with either nebulized IFN α2b and umifenovir or a combination of both. Treatment with IFN α2b (with or without umifenovir) significantly reduced the duration of detectable virus in the upper respiratory tract and the duration of elevated inflammatory markers. The study had limitations, which are the absence of a control group and the exclusive enrollment of moderate forms of COVID-19. IFN β1a in combination with hydroxychloroquine and lopinavir/ritonavir was effective in reducing disease symptoms at a low dose of 44 μg administered subcutaneously every other day up to 10 days [62]. A trial in China (ChiCTR2000029308) has been registered for hospitalized COVID-19 patients, estimating the efficacy of ritonavir-lopinavir and IFN α2b combined therapy [1,16].

Further, Chinese physicians have used steroids to control cytokine storm related to the infection and occurrence of lung fibrosis. However, steroids have a narrow therapeutic window since these should be used only when SARS-CoV2 has been already eliminated by the human immune system. Otherwise, virus replication and shedding and related symptoms of the infection may exacerbate [59].

The utility of combined administration of IVIgG (intravenous Immunoglobulin G) and low molecular weight heparin (LMWH) anticoagulant therapy was suggested since infection, inflammation, and other factors may lead to disseminated intravascular coagulation [14].

Chloroquine (CQ) and hydroxychloroquine (HCQ) have been used for prevention and treatment of malaria [63]. They have immunomodulatory, rather than suppressive, characteristics that were revealed valid in the management of rheumatoid arthritis and lupus erythematosus. These drugs apparently act by preventing antigen processing and presentation to the Major Histocompatibility Complex (MHC) class II molecules due to increased pH and inhibition of lysosomal activities in antigen presenting cells [64]. As a consequence, T cell activation is compromised, as well as the expression of costimulatory molecules by T and B lymphocytes and secretion of proinflammatory cytokines (i.e., IL1, IL6, TNF) [65]. The drug blocks binding of Toll-like receptor (TLR) and DNA/RNA ligand; therefore, it blocks the TLR signaling cascade and the binding of cytosolic DNA with cGAMP (cyclic guanosine monophosphate–adenosine monophosphate) synthase, thus inhibiting cytokines secretion [66,67,68,69,70]. Furthermore, evidence is provided for chloroquine-wide antiviral activities; the drug indeed increased endosomal pH requested for virus/cell fusion and interfered with the glycosylation of cellular receptors for SARS-CoV [63,70]. A number of clinical trials conducted in China demonstrated apparent efficacy and acceptable safety of chloroquine or hydroxychloroquine in the treatment of COVID-19 associated pneumonia [70]. Regarding potential side effects, CQ and HCQ are not immunosuppressant; therefore, they are not without risk of infectious complications. Both can produce gastrointestinal side effects of vomiting and diarrhea. Long-term treatment with CQ and HCQ can produce retinopathy and cardiomyopathy [71]. HCQ has a lower level of tissue accumulation; therefore, its usage is associated with lower adverse effects [64].

Teicoplanin is another drug under investigation for COVID-19 infection. This is a glycopeptide antibiotic, currently used in the treatment of Gram-positive bacterial infection, especially in Staphylococcal infections. Already, this antibiotic showed antiviral activity in vitro against viruses, including Ebola, influenza, flavivirus, hepatitis C, HIV, MERS-CoV, and SARS-CoV [72,73,74]. Teicoplanin would act on the early step of genomic viral RNA release and the continuation of SARS-CoV2 life cycle by blocking the low pH cleavage by cathepsin L of the spike protein in the late endosomes [75]. These preliminary results should be confirmed by randomized clinical trials [72].

## 4. Prophylaxis

### Vaccines

The emergency to combat the COVID-19 pandemic has propelled the search for vaccine development in the scientific community. The urgent need for a vaccine raises from several concerns, which are not only control of the infection in its spreading from its real geographical origin in Wuhan and the whole country of China, but also worldwide with the consequent disruption of the social and economic equilibrium, especially in countries with poor healthcare resources [18]. Further, there could be the necessity to put under control cluster cases that may represent in the near future.

In the past 10 years, approaches were already attempted to combat CoV viruses [16,77]. Surface spike glycoproteins or S-proteins have been principally considered optimal targets for raising neutralizing antibodies and T cell responses to this virus family [16]. S-protein-based vaccines should generate antibodies against viral binding to ACE2 but also virus uncoating [49]. S-proteins were employed as full-length proteins or in their most immunogenic parts. Recombinant S-protein-based strategies have also been attempted by using expression in virus-like particles (VLP), DNA, or viral vectors [16,30,49,77,78,79]. It was suggested that the S1-receptor-binding domain has a superior capacity to induce neutralizing antibodies [78].

As for previous attempts in vaccine preclinical experimentation to SARS-CoV, virus studies are needed to ascertain the most suitable animal models, especially electing small animals that closely resemble the clinical disease caused by SARS-CoV2 infection in humans and its etiopathogenetic mechanisms [16]. These are at low cost and of easy manipulation, and they would present receptor affinity for the virus and generate specific immune responses and protection [16].

Regarding the present pandemic, immunoinformatics databases can support the identification of strategic target epitopes, promising candidates for immune recognition to be used in COVID-19 vaccine developments. Indeed, effective binding of identified epitopes for cytotoxic T lymphocytes (CTL) responses and the peptide binding grooves on MHC class I molecules was proven, indicating their potential to generate immune responses [80,81].

It is estimated that more than 100 vaccines are currently being produced (https://www.darwinresearch.com/astrazeneca-ramps-up-covid-19-vaccine-manufacturing-distribution-capacity-with-two-more-deals, accessed on June 2020) [82]. These platforms explore different approaches based on S glycoprotein as the major inducer of neutralizing antibodies. Thus, full-length or appropriate parts of the S glycoprotein are believed to be the most promising candidate vaccine for previous SARS-CoV viruses attempts. S-protein immunogenicity or its binding to ACE2 receptor, critical for virus access into host cells, is apparently not affected by the presence or absence of other structural viral proteins [16].

The immunity generated by CoV2 vaccines is currently assessed in preclinical studies and started trials through humoral antibody induction and/or T cell effector responses [83]. Of note, vaccines that do not elicit a skewed Th2 immune response are considered ideal for controlling SARS-CoV2 infection since the generation of a skewed Th2 T cell response involving IL-10 and IL-4 cytokines would suppress inflammatory CD8+ T cells contributing to exacerbate pathology [83,84]. In this regard, live attenuated vaccines (LAV) employ viruses that are rendered replication-incompetent through different passages in culture [83] and inactivated vaccines employ pathogen been killed throughout exposure to chemicals and heat. At present, Codagenics is proposing a computationally designed immunogenic, but not pathogenic, SARS-CoV2 virus. SinoVac has published preclinical data regarding an inactivated purified SARS-CoV2 vaccine (PiCoVacc) [85] that induced specific neutralizing antibodies in mice, rats, and non-human primates. These antibodies showed broader neutralizing activity against 10 representative SARS-CoV strains. When administered using two different doses (3 μg and 6 μg) to a macaque model, partial or complete protection was obtained; this was mediated by humoral immunity with controlled T cell responses to avoid immunopathology [85]. Protein subunit vaccines are produced in vitro by employing antigenic proteins that induce a protective immune response, avoiding the use of highly pathogenic live particles (Generex Biotechnology, Vaxart, Medicago, GSK, and Clover Biopharmaceuticals, University of Queensland) [83]. These predominantly elicit a humoral antibody response by administration with an adjuvant.

Several nucleic acid vaccines are currently under development for COVID-19 [83]. Recombinant viral vector vaccines utilize the backbone from a genetically modified virus, such as adenoviruses, poxviruses, and Vesicular stomatitis virus, having insertion sites for genes of the target pathogen, which becomes expressed intracellularly in the host upon vaccination [83]. This approach may encounter the generation of immunity toward the vector that could hinder the vaccine specific response after boost vaccination. Further, human adenoviruses (hAds) can circulate at high frequency, thus pre-existing immunity may reduce vaccine efficacy. In this regard, chimpanzee adenoviruses can elicit a similar or superior immunogenicity to hAds. Imophoron at Bristol University is developing an adenovirus-based SARS-CoV2 VLP vaccine (ADDomer© VLP, Bristol, UK) at present in preclinical assessment which presents rapid development and self-adjuvant characteristics without cold-storage requirement (https://www.bristol.ac.uk/news/2020/april/codid-19-vaccine-platform.html, accessed on June 2020). In the UK, the Jenner Institute and the Oxford vaccine group (https://covid19vaccinetrial.co.uk, accessed on June 2020) has produced the ChAdOx1 nCOV-19 vaccine that employs the replication-deficient chimpanzee adenoviral vector isolate Y25. This vectored vaccine encodes a codon-optimized full-length S-protein with a human tPA (tissue plasminogen activator) leader sequence. Preclinical studies have already demonstrated that it is immunogenic in mice by eliciting a strong humoral and not Th2 dominated cell-mediated response. A single vaccination was effective in rhesus macaques. A reduced viral load was observed in bronchoalveolar lavage and respiratory tract of vaccinated animals respect to controls [86]. This vaccine has recently entered a Phase I/II clinical trial, already involving 900 volunteers aged 18–55 that received 5 × 10^10^ viral particles (clinicaltrials.gov: NCT04324606). Vaccination with a lipid nanoparticle-encapsulated mRNA-1273 encoding SARS-CoV2 S-protein developed by Moderna (NCT042283461) [83,85] prevented viral replication in the lungs of mice challenged with CoV2. Neutralizing titers in Phase 1 trial participants receiving 2 µg and 100 µg doses were consistent with those protective in the mouse challenge model. Phase I/II trials are currently ongoing (https://investors.modernatx.com/news-releases/news-release-details/moderna-announces-positive-interim-phase-1-data-its-mrna-vaccine/, accessed on June 2020). Adenovirus type 5 vector expressing S-protein (Ad5-nCoV) from CanSino Biological (NCT04313127) is in Phase I. Participants with pre-existing Ad5 neutralizing antibodies exhibited lower humoral and cellular responses. BNT162 (produced by BioNTech, Mainz, Germany) employed a modified RNA delivered in a lipid nanoparticle [83]. The bacTRL platform exploited by Symvivo Corporation (Canada) utilizes engineered probiotic *Bifidobacterium longum* to deliver CoV2 S-protein into intestinal cells (NCT 04334980) [83]. Among the other candidates, INO-4800 produced by Inovio Pharmaceuticals (NCT04336410) is based on a DNA plasmid encoding S-protein [87]. Preclinical studies have assessed immunization of mice and guinea pigs with INO-4800 measuring antigen-specific T cell responses, functional antibodies that neutralize the SARS-CoV2 infection, and block binding of spike glycoprotein to ACE2 receptors. Presence of SARS-CoV2 antibodies in the lungs was also demonstrated [88]. Shenzhen Geno-Immune Medical Institute has generated dendritic cells (DCs) (LV-SMENP-DC in Phase 1, NCT042276896) and artificial APCs (aAPCs) modified with a lentiviral vector expressing a synthetic minigene based on parts of selected viral proteins (pathogen-specific aAPC, Phase I, NCT04299724).

## 5. Conclusions

SARS-CoV2 has recently emerged as a novel unknown virus. Today, we are facing the health and economic consequences of its rapid diffusion worldwide. In a guarded optimistic view, we hope that the undertaken preventive and treatment measures will at least contribute to contain diffusion, attenuate activity or even eliminate the virus. Although today there are no specific treatments for COVID-19, early diagnosis and improved management of severe patients may reduce mortality. As above underlined an effective measure is to treat a cytokine storm by administering anti-inflammatory drugs, such as tocilizumab, at early stage of the infection for timely control of the disease and to avoid the risk of mortality. Modern technologies may subside in a near future to validate vaccination strategies in eradicating viral infection from humans. In this regard, reverse vaccinology based on computational identification of immunodominant epitopes may contribute to novel preventive strategies for COVID-19. In providing examples, indeed, epitope-based immune-derived vaccines (IDV) are generally considered to be safe when compared to other vectored or attenuated live vaccines. These may also provide essential T-cell help for antibody-directed vaccines [89]. Several platforms have being explored; results on their preclinical efficacy due to generated humoral T cell and reduced viral load responses are already available. Development and optimization of recent technologies, such as multimer staining for flow cytometry analysis, offer potential in characterizing COVID-19-specific HLA class I and II CD8+ and CD4+ T cell responses and eventually correlate their frequency with disease severity and outcome. This may open the pathway to novel diagnostic strategies [90,91]. Random peptide library screening with CoV2 monoclonal antibodies may also lead to the identification of immunodominant epitopes for vaccine design [92]. Further, combinatorial antibody phage display may be empowered to identify the immune repertoire of SARS-CoV2-specific neutralizing antibodies. As suggested for other viral infections [93], this approach may lead to elucidate mechanisms governing viral infection and clearance at the different stages of disease and advance effective immunotherapies based on monoclonal antibodies discovery.

## Figures and Tables

**Table 1:** Present Coronavirus (CoV) drug treatment options.

Treatment (Therapeutics Class)	Target/Mechanism	Reference
Small molecules inhibitors	Helicase	Patents [50]
		KR20100029528A; 2010
		KR20110006083A; 2011
	Proteases	Patents [50]
		KR20100066142A; 2010
		CN101418334A; 2009
		CN101701245A;2010
		CN101921823A; 2010
		KR20110068191A; 2011
	Host cell proteases	[16,18,50]
	Host cell endocytosis	[16,18,50]
	S-protein/Inhibition of SARS-CoV fusion and entry into the host cell	[49]
	S cleavage/Inhibition of functional S1 and S2 subunits production	[49]
	RBD-ACE2/Blocking S-protein-mediated infection	[49]
	RNA synthesis inhibitors (3TC, TDF)	[18]
Renin-angiotensin system (RAS) inhibitors	ACE1 and Angiotensin type 1 receptors	[54]
Antisense RNA (siRNA)	Reduction of virus replication and/or silencing of S gene expression	[49]
		Patents [50]
		CN101173275; 2008
Ribozyme	Cleavage of coronavirus gene	Patents [50]
		US2010273997A1;2010
Neutralizing antibodies	Spike glycoprotein/Inhibit SARS-CoV fusion and entry into the host cell	[16,49]
IVIgG and LMWH (low molecular weight heparin) anticoagulant therapy	Inhibition of biological activity/viral replication	[14,16]
mAbs	Full-length S-protein orS1-receptor-binding domain (RBD)/Inhibition of SARS-CoV fusion and entry into the host cell	[16]
Antiviral peptides acting on S2	Inhibition of production of functional S2	[16]
Remdesivir (Nucleoside analogues)	RdRp/Terminates the non-obligate chain	[45,52]
Lopinavir/Ritonavir/ASC09F/ Darunavir/Cobicistat	3CLpro/Protease inhibitors	[52]
Oseltamivir	Neuraminidase inhibitors	[50]
Peptide EK1	Spike glycoprotein/Inhibits pan-CoV fusion	[50,76]
Arbidol (Antiviral)	Effect on several stages of the viral life cycle, such as cell entry (attachment, internalization) and replication	[16]
Association Lopinavir/Ritonavir/Ribavirin/ IFN beta1b	SARS anti-CoV2 activity	[60]
Association IFN alpha2b/Umifenovir	SARS anti-CoV2 activity	[61]
Association IFN beta1a hydroxychloroquine Lopinavir/Ritonavir	SARS anti-CoV2 activity	[62]
Chloroquine and hydroxychloroquine	Endosomal acidification/Acting on a lysosomotropic base that appears to disrupt intracellulartrafficking and viral fusion events	[64,70]
Teicoplanin (glycopeptide antibiotic)	Blocked virus entry by specifically inhibiting the activity of cathepsin L	[72,75]
Tocilizumab (recombinant humanized anti-human IL-6 receptor monoclonal antibody)	IL-6 blocker	[57,58]

## Abbreviations

aAPCs	Artificial antigen-presenting cells
ACE2	Angiotensin Converting Enzyme 2
Ad5	Adenovirus 5
ALT	Alanine aminotransferase
ARDS	Acute-respiratory disease syndrome
AST	Aspartate aminotransferase
AT1R	Angiotensin type-1 receptor
CAR	Chimeric antigen receptor
CEPI	Coalition for Epidemic Preparedness Innovations
cGAMP	Cyclic guanosine monophosphate–adenosine monophosphate
CoV	Coronavirus
COVID-19	Coronavirus disease 2019
CP	Convalescent plasma
CQ	Chloroquine
CRP	C reactive protein
CRS	Cytokine release syndrome
CTL	Cytotoxic T lymphocytes
DCs	Dendritic cells
ESR	Erythrocyte sedimentation rate
hAds	Human adenoviruses
HCQ	Hydroxychloroquine
HIV	Human Immunodeficiency Virus
IDV	Immune-derived vaccines
iPSC	Induced pluripotent stem cells
IVIgG	Intravenous Immunoglobulin G
LAMP	Loop-mediated isothermal amplification
LAV	Live attenuated vaccines
LDH	Lactate dehydrogenase
LMWH	Low molecular weight heparin
mAbs	Monoclonal antibodies
MDW	Monocyte volume distribution width
MERS	Middle East Respiratory Syndrome
MHC	Major Histocompatibility Complex
PRR	Pattern Recognition Receptors
PT	Prothrombin time
RAS	Renin–angiotensin system
RBD	Receptor binding domain
RdRp	RNA-dependent RNA polymerase
rRT-PCR	Reverse real-time PCR
SARS	Severe Acute Respiratory Syndrome
sdAb	Single-domain antibodies
siRNAs	Anti-sense RNA
TLR	Toll like receptor
tPA	Tissue plasminogen activator
VLP	Virus-like particles
